# Safety of repeated use of emergency contraceptive pills in the same menstrual cycle: a systematic review

**DOI:** 10.1136/bmjsrh-2025-202841

**Published:** 2025-11-05

**Authors:** Petrus Schonken Steyn, Erin Fleurant, Emma Marie Smith, James N Kiarie

**Affiliations:** 1 UNDP/UNFPA/UNICEF/WHO/World Bank Special Programme of Research, Development and Research Training in Human Reproduction, Department of Sexual and Reproductive Health and Research, World Health Organization, Geneva, Switzerland; 2 Division of Complex Family Planning, Department of Obstetrics and Gynecology, Endeavor Health, Evanston, Illinois, USA; 3 Department of Obstetrics & Gynecology, Complex Family Planning, Kaiser Permanent Medical Centre, Oakland, California, USA

**Keywords:** contraceptives, postcoital, Patient Safety

## Abstract

**Background:**

Emergency contraception refers to contraceptive methods used following unprotected sexual intercourse.

**Objective:**

To review the literature on safety of repeated use of emergency contraceptive pills (ECPs) in the same menstrual cycle.

**Methods:**

We searched multiple databases from inception through February 2024 for articles on repeated ECP use. We assessed the risk of bias for each study and certainty of evidence for all outcomes.

**Results:**

From 4565 articles identified, six met the inclusion criteria. Four studies of repeated levonorgestrel (LNG)-ECP use provided very low certainty evidence. Among women who became pregnant after at least one use of LNG-ECP in the conception cycle, women with ectopic pregnancy had increased odds of repeated LNG-ECP use in the conception cycle compared with women with intrauterine pregnancy (adjusted OR (aOR) 2.5, 95% CI 1.0 to 6.2). One non-comparative study reported few serious adverse events with repeated pericoital use of LNG-ECP, and two cohort studies of LNG-ECP failure found no differences in pregnancy, fetal/neonatal, infant or child outcomes comparing higher (2.25–9 mg LNG) and lower (0.75–1.5 mg LNG) doses in the conception cycle. Two studies of repeated ulipristal acetate (UPA) use provided very low certainty evidence. One non-comparative study observed no serious adverse events, no abnormal laboratory results and normal endometrial biopsies with UPA (30 mg, 4–6 doses/month). One randomised controlled trial in a clinical setting observed no serious adverse events with UPA (10 mg, 20 mg or 50 mg for 10 days) compared with placebo.

**Conclusion:**

Evidence on the safety of repeated ECP use is limited and of very low certainty, but overall does not suggest safety concerns.

**WHAT IS ALREADY KNOWN ON THIS TOPIC**

- Emergency contraceptive pills (ECPs) are safe to use, should be taken as soon as possible within 5 days after unprotected sexual intercourse (UPSI), and can be provided in advance so they are available when needed.
- The WHO Medical Eligibility Criteria for Contraceptive Use (WHO MEC) provides recommendations for the safe use of ECPs for women with medical conditions or personal characteristics, including repeated use of ECPs. A previous systematic review of the safety of ECPs identified one study on repeated use and found that multiple doses of levonorgestrel (LNG)-ECPs over 1 year were not associated with ectopic pregnancy. However, that review did not address the safety of ECP use more than once in the same menstrual cycle.

**WHAT THIS STUDY ADDS**

- The objective of this review was to summarise the literature on the safety of repeated use of ECPs in the same menstrual cycle, as part of the process to update the WHO MEC.

**HOW THIS STUDY MIGHT AFFECT RESEARCH, PRACTICE OR POLICY**

- Evidence on the safety of repeated ECP use is limited and of very low certainty, but overall does not suggest safety concerns, thus repeated use should not be discouraged during patient-centred care and counselling to prevent unintended pregnancies when multiple acts of UPSI are encountered during one menstrual cycle.

## Introduction

 Emergency contraception, or postcoital contraception, refers to contraceptive methods used following unprotected sexual intercourse (UPSI), including after lack of contraceptive use, contraceptive misuse (such as forgotten pills or torn condoms) and sexual assault.^1^ There are three emergency contraceptive pill (ECP) methods included in the WHO Medical Eligibility Criteria for Contraceptive Use (WHO MEC) and WHO Selected Practice Recommendations for Contraceptive Use (WHO SPR): a combined oral contraceptive (COC) regimen, oral levonorgestrel (LNG; 1.5 mg, either as a single dose or two 0.75 mg doses taken 12 hours apart) and ulipristal acetate (UPA; 30 mg).^2 3^ ECPs are safe to use, should be taken as soon as possible within 5 days after UPSI, and can be provided in advance so they are available when needed.^2 3^

The WHO MEC provides recommendations for the safe use of ECPs for women with medical conditions or personal characteristics, including repeated use of ECPs.^2^ A previous systematic review of the safety of ECPs identified one study on repeated use and found that multiple doses of LNG-ECPs over 1 year were not associated with ectopic pregnancy.^4 5^ However, that review did not address the safety of ECP use more than once in a single menstrual cycle. The objective of this review was to summarise the literature on the safety of repeated use of ECPs in the same cycle, as part of the process to update the WHO MEC. We focused on the research question: Among women using ECPs, does taking repeated doses of ECPs (ideally within one menstrual cycle) compared with taking only one or no dose of ECPs increase risk of adverse events?

## Methods

We conducted this systematic review according to an a priori protocol, which has been registered in the International Prospective Register of Systematic Reviews (PROSPERO) under the registration number CRD42024507789^6^ and reported in accordance with the Preferred Reporting Items for Systematic Reviews and Meta-Analyses (PRISMA) guidelines.^7^

### Eligibility criteria

We included studies that assessed safety outcomes associated with repeated use of ECPs. Study participants included women of reproductive age (eg, 15–49 years, but ranges may differ based on inclusion criteria in specific studies). The exposure was repeated doses of ECPs within a defined time frame (ideally within one menstrual cycle but longer time intervals could be accepted). We included ECP methods and doses described in the WHO MEC and SPR, including COCs, LNG and UPA.^2 3^ We accepted studies that included ECPs used as emergency contraception (taken after UPSI), as pericoital contraception (taken around the time of UPSI) or some other regimen. We also accepted studies that examined ECP doses similar to those described in the WHO MEC and WHO SPR. The comparison was a single ECP dose or no ECP use. Outcomes included adverse health events (eg, thrombosis), side effects (eg, bleeding irregularities) and patient satisfaction among study participants, as well as outcomes among ECP users who became pregnant and therefore may have been exposed to ECP use in early pregnancy (eg, pregnancy, fetal/neonatal, infant or child outcomes). We included primary data reported in published randomised controlled trials (RCTs) in a clinical setting, non-randomised clinical trials (comparative and non-comparative), cohort studies (comparative and non-comparative), case–control studies and cross-sectional studies that included a retrospective analysis of changes in outcomes over time. We excluded cross-sectional studies, case reports, commentaries and reviews.

### Literature search

We worked with a research librarian to develop a comprehensive search strategy (online supplemental appendix 1). The search was conducted in MEDLINE, EMBASE, Cochrane Library, CINAHL and ClinicalTrials.gov databases from database inception to 28 February 2024. We also reviewed references in systematic reviews and key review articles to optimise our search. We considered articles in all languages.

### Study selection and data extraction

Two coauthors independently screened titles and abstracts from the search and identified full-text articles to be reviewed to determine eligibility using Covidence.^8^ Two coauthors then reviewed full-text articles to determine whether they met our inclusion criteria. Any conflicts were resolved by coauthor discussion. We used standardised evidence tables to extract information from each study.

### Risk of bias assessment

We used the Cochrane risk of bias tool to assess risk of bias in randomised trials and a modified version of the Cochrane risk of bias tool to assess risk of bias in non-randomised studies.^9 10^ Two coauthors independently assessed the risk of bias of each included study, and any discrepancies were resolved through discussion.

### Data synthesis

We summarised descriptive statistics, unadjusted and adjusted point estimates, results of significance tests and interval estimates. We did not conduct meta-analyses due to the heterogeneity of study populations, exposures and outcomes.

### Certainty of evidence

We assessed the certainty of evidence for all outcomes using the Grading of Recommendations Assessment, Development and Evaluation (GRADE) system.^11 12^ For each outcome, we rated several elements (risk of bias, inconsistency, imprecision and indirectness) as not serious, serious or very serious. Indirectness refers to a study population, exposure or outcome that provides indirect information about the study question. Based on these assessments, the certainty of evidence was rated as high, moderate, low or very low. RCTs are considered to start at high certainty and observational studies at low certainty; ratings are then adjusted based on the elements previously listed.

## Results

**Figure 1:**
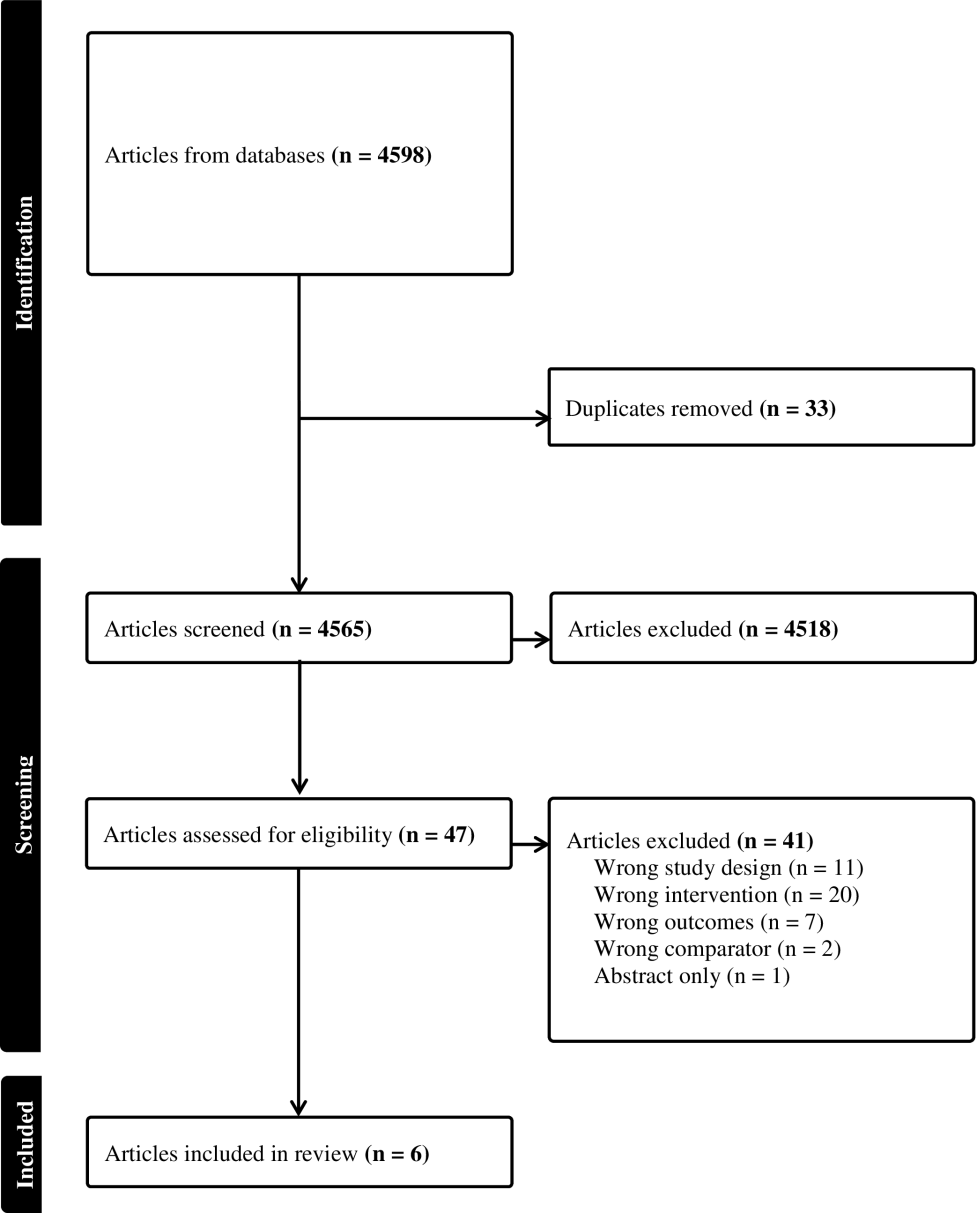
PRISMA (Preferred Reporting Items for Systematic Reviews and Meta-Analyses) flowchart of the search process for evidence on the safety of repeated use of emergency contraceptive pills.

From 4598 abstracts identified by the search, 33 were duplicates and 4518 were not relevant to the review question (figure 1). We screened 47 full-text studies and excluded 41, primarily due to wrong intervention or wrong study design. Six studies met the inclusion criteria.^513^,^17^ Studies were of varied designs, including one RCT, two non-comparative trials, two cohort studies and one case–control study. Four studies provided evidence for the safety of repeated use of LNG^5 13 16 17^ and two for UPA.^14 15^

**Figure 2:**
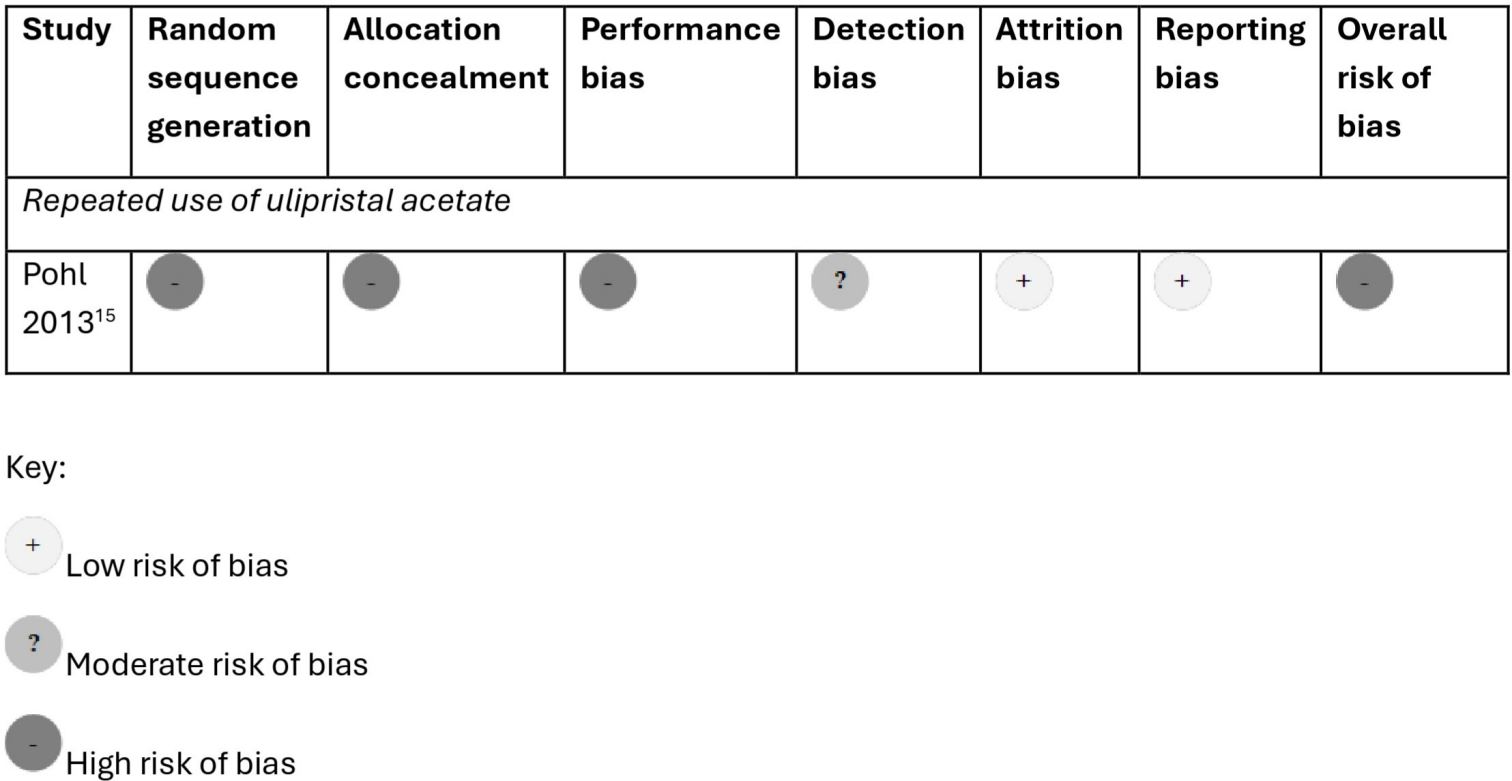
Risk of bias in studies on the safety of repeated use of emergency contraceptive pills: randomised controlled trials.

The risk of bias was high for all six studies^513^,^17^ (figures2  3). For the only RCT,^15^ there was inadequate information about random sequence generation, allocation concealment, and blinding of participants and outcome assessors. For the non-randomised trial and observational studies, high risk of bias was primarily due to unclear or inadequate assessment of the exposure, comparison group and adverse health events, and lack of assessment for the potential for confounding in comparative studies.^5 13 14 16 17^

**Figure 3:**
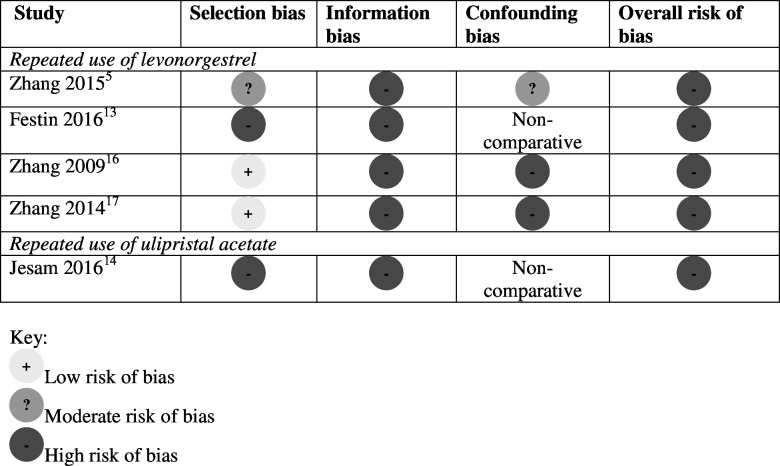
Risk of bias in studies on the safety of repeated use of emergency contraceptive pills: non-randomised studies.

Four studies provided evidence for safety of repeated use of LNG-ECPs (table 1).^5 13 16 17^ One case–control study evaluated the association between repeated use of LNG within a single ovulatory cycle and risk for ectopic pregnancy.^5^ The study included 2411 women with ectopic pregnancies compared with 2416 women with intrauterine pregnancies. Among women who became pregnant after at least one use of LNG in the conception cycle, women with ectopic pregnancy had increased odds of repeated LNG in the conception cycle compared with women with intrauterine pregnancy (adjusted OR (aOR) 2.5, 95% CI 1.0 to 6.2). A non-comparative trial assessed safety of pericoital use of LNG 1.5 mg pills (within 24 hours before or after UPSI) among 330 women over 6.5 months of follow-up.^13^ Participants used a mean of four to seven pills per month. Among 102 reported adverse events, three (2.9%) events were severe and judged to be unrelated to LNG use (choledocholithiasis and subsequent surgery in one participant; ruptured corpus luteum cyst in another participant), 19.6% were moderate and 77.5% were mild; moderate and mild adverse events included headache, nausea, and abdominal and pelvic pain. Vaginal bleeding patterns were generally lighter over follow-up and rates of anaemia decreased. Two cohort studies with overlapping study populations assessed the association between LNG exposure during the conception cycle and subsequent pregnancy, fetal/neonatal and infant/child outcomes.^16 17^ The first of these cohort studies assessed pregnancy and fetal/neonatal outcomes (ie, ectopic pregnancy, spontaneous abortion, second-trimester abortion with fetal abnormalities, preterm delivery, stillbirth, low birth weight (<2500 g), fetal or neonatal malformation, and pregnancy complications).^16^ LNG exposure during the conception cycle was categorised as high dose (>1.5 mg; 74% in this group reported 3 mg cumulative use, double the typical dose of LNG as ECP), low dose (≤1.5 mg; 94% in this group reported 1.5 mg use, the typical dose of LNG as ECP) or no exposure. The primary comparison for the study was any LNG (n=301) versus no LNG (n=325) exposure; there were no statistically significant differences in any of the outcomes (all p>0.05). The primary outcome of interest for this review was the comparison of high-dose LNG-ECP (n=33) versus low-dose LNG-ECP (n=268). The proportions of those who experienced adverse outcomes appeared similar in the high-dose and low-dose groups for all outcomes; statistical testing was not reported. The second cohort study included a subset of those enrolled in the previous study and assessed infant and child development outcomes at 3, 6, 12 and 24 months of age (ie, height, weight, head circumference, intelligence scores).^17^ In this cohort, 172 participants were in the low-dose LNG group, 19 were in the high-dose LNG group and 211 were in the non-exposed group. While the low-dose and high-dose groups were not directly compared, there were no differences across the low-dose, high-dose and non-exposed groups in weight (p=0.061), height (p=0.350), head circumference (p=0.308), development quotient (p=0.476) and mental index (p=0.872) at any time points.

**Table 1:** Evidence table for the safety of repeated use of emergency contraceptive pills

Authors, year, country, funding source	Study design	Population	Intervention	Comparison	Outcomes	Results
Repeated use of LNG-ECP						
Zhang *et al*, 2015 China Shanghai Scientific and Technical Committee Grants^5^	Case–control	Cases: 2411 women with EP Controls: 2416 women with IUP Controls: 2419 women with no pregnancy	LNG-ECP (1.5 mg), repeated use with further acts of intercourse in same cycle Mean doses per cycle NR	LNG-ECP (1.5 mg), single use in same cycle with no further acts of intercourse in same cycle	EP	aOR (95% CI) EP vs IUP: 2.5 (1.0 to 6.2), 14%* vs 5%* EP vs no pregnancy: 3.1 (1.1 to 8.7), 14%* vs 5%*
Festin *et al*, 2016 Brazil, Hungary, Singapore, Hungary UNDP/UNFPA/ UNICEF/WHO/World Bank Special Programme, Gynuity, Bill and Melinda Gates Foundation^13^	Non-randomised, non-comparative trial	330 women, aged 18–45 years	LNG-ECP (1.5 mg), pericoital use (24 hours before or after intercourse) Mean 4–7 doses per month	N/A	AEs Vaginal bleeding patterns and anaemia Acceptability Follow-up: 6.5 months	Total AEs: 102 Severe AEs: 3 events (2.9%) (choledocholithiasis and surgery in the same participant; ovarian cyst) Moderate AEs: 20 events (19.6%) Mild AEs: 79 events (77.5%) Light vaginal bleeding generally increased and very heavy vaginal bleeding generally decreased over time and with greater number of pills use Moderate anaemia (8–10 g/dL) decreased from 1.5% at baseline to 0.8% at follow-up; one case of severe anaemia was identified at the final visit
Zhang *et al*, 2009 China Shanghai Population and Family Planning Commission, Shanghai Young University Teachers Special Fund^16^	Cohort	626 pregnant women LNG-ECP exposure during conception cycle (n=301) No LNG-ECP exposure during conception cycle (n=325)	LNG-ECP (>1.5 mg) during conception cycle (n=33) Cumulative LNG dose: 2.25–9 mg, mostly 3 mg (74%)	LNG-ECP (≤1.5 mg) during conception cycle (n=268) LNG dose: 0.75–1.5 mg, mostly 1.5 mg (94%)	Adverse pregnancy, fetal and neonatal outcomes	LNG-ECP >1.5 mg vs ≤1.5 mg EP: 0% vs 0.4% SAB: 9.1% vs 10.4% Second-trimester abortion (fetal abnormalities): 0% vs 0.4% PTD: 3.3% vs 5% Stillbirth: 0% vs 0.4% Low birth weight (<2500 g): 3.3% vs 2.5% Malformation (fetal or neonatal): 0% vs 1.5% Pregnancy complications: 25.0% vs 30.5% Statistical testing NR for comparisons by LNG dose LNG-ECP (all doses) vs no LNG No statistically significant difference in outcomes (all p>0.05)
Zhang *et al*, 2014 China Shanghai Population and Family Planning Commission, Shanghai Young University Teachers Special Fund^17^	Cohort	402 women with 409 infants LNG-ECP exposure in pregnancy (n=191 women) No LNG-ECP exposure in pregnancy (n=211 women)	LNG-ECP (>1.5 mg) during conception cycle (n=19 women)	LNG-ECP (≤1.5 mg) during conception cycle (n=172 women)	Infant/child physical and mental development (height, weight, head circumference, intelligence scores) Follow-up: 3, 6, 12 and 24 months of neonatal age	LNG-ECP >1.5 mg vs ≤1.5 mg vs no LNG Weight (p=0.061) Height (p=0.350) Head circumference (p=0.308) Development quotient (p=0.476) Mental index (p=0.872)
Repeated use of UPA						
Jesam *et al*, 2016 Chile, Dominican Republic HRA Pharma^14^	Non-randomised, non-comparative trial	23 women, aged 18–35 years with history of tubal ligation	UPA (30 mg): Every 7 days for 8 weeks (n=12), 4 doses per month Every 5 days for 8 weeks (n=11), 6 doses per month	NA	AEs Laboratory parameters (CBC, LFTs, electrolyte profile, blood glucose, lipid, VTE markers) Endometrial biopsy assessment Follow-up: 18–23 days after the first post-treatment menses	Serious AEs: 0 Mild/moderate AEs: 68 among 22 (95.7%) participants (headache, nasopharyngitis, influenza and mild anaemia) No clinically important changes in laboratory parameters, including VTE markers All biopsies benign with no hyperplasia; no changes in endometrial thickness
Pohl *et al*, 2013 France Preglem SA/Gideon Richter^15^	RCT	32 women, aged 18–45 years	UPA, daily for 10 consecutive days: 10 mg (n=8) 20 mg (n=8) 50 mg (n=8)	Placebo, daily for 10 consecutive days (n=8)	AEs Follow-up: up to 14 days after last UPA dose	Serious AEs: 0 Mild/moderate AEs: UPA 10 mg: 37.5% UPA 20 mg: 50.0% UPA 50 mg: 62.5% Placebo: 62.5%

* Event rates (ectopic pregnancy, intrauterine pregnancy, no pregnancy) among those with repeated LNG-ECP use.

AE, adverse event; aOR, adjusted odds ratio; CBC, complete blood count; EC, emergency contraception; ECP, emergency contraceptive pill; EP, ectopic pregnancy; IUP, intrauterine pregnancy; LFT, liver function test; LNG, levonorgestrel; NA, not applicable; NR, not reported; PTD, preterm delivery; RCT, randomised controlled trial; SAB, spontaneous abortion; UPA, ulipristal acetate; VTE, venous thromboembolism.

Two studies provided evidence for safety of repeated use of UPA (table 1).^14 15^ A non-comparative study of 23 participants assessed repeated use of UPA 30 mg either every 7 days or every 5 days for eight consecutive weeks.^14^ While no serious adverse events were noted, 95.7% of participants reported mild or moderate adverse events, including headache, nasopharyngitis, influenza and mild anaemia. Laboratory findings were normal, including complete blood count, liver function tests, electrolyte, blood glucose, lipid and venous thromboembolism markers. All endometrial biopsies were benign with no hyperplasia, and there were no changes in endometrial thickness. One trial randomised 32 participants to daily use of UPA at different doses (placebo, 10 mg, 20 mg and 50 mg) for 10 days.^15^ No serious adverse events were reported. Reports of mild/moderate adverse events among the placebo group (62.5%) were similar to the UPA 50 mg group (62.5%), with decreased reports for lower UPA doses (UPA 20 mg (50.0%) and UPA 10 mg (37.5%)).

Certainty of evidence was determined to be very low for all outcomes for both LNG- and UPA-ECPs. Among four studies that assessed outcomes associated with repeated use of LNG, study designs included two cohort studies,^16 17^ a case–control study^5^ and a non-comparative trial.^13^ All had very serious concerns for risk of bias, inconsistency could not be assessed as there was only one study for each outcome assessed, imprecision ranged from serious to very serious concern, and two of the four studies provided indirect evidence. For UPA, both the RCT^15^ and the non-comparative trial^14^ had very serious concerns for risk of bias and imprecision, inconsistency could not be assessed, and one study provided indirect evidence.

## Discussion

We identified four studies that provided evidence on the safety of repeated use of LNG as ECPs.^5 13 16 17^ Evidence from one case–control study suggested that women with ectopic pregnancy had increased odds of repeated LNG use in the conception cycle compared with women with intrauterine pregnancy; however, the confidence intervals were wide and included 1.0.^5^ One non-comparative study reported few serious adverse events with repeated pericoital use of LNG,^13^ and two cohort studies of LNG failure found no differences in pregnancy, fetal/neonatal, infant or child development outcomes comparing higher (2.25–9 mg LNG) and lower (0.75–1.5 mg LNG) doses.^16 17^ Two studies assessed repeated UPA use.^14 15^ One non-comparative study observed no serious adverse events, no abnormal laboratory results and normal endometrial biopsies with UPA (30 mg, 4–6 doses/month).^14^ One RCT observed no serious adverse events with UPA (10 mg, 20 mg or 50 mg for 10 days) compared with placebo.^15^

There are several limitations to consider with this body of evidence. Of the six studies, only one was an RCT,^15^ three were comparative observational studies^5 16 17^ and two did not include a comparison group,^13 14^ making the results difficult to interpret. All studies had a high risk of bias, further challenging interpretation. Very serious concerns for bias and imprecision, along with lack of ability to assess inconsistency (as there was only one study for each outcome) and indirectness of three studies, led to the determination that the evidence was of very low certainty for all outcomes. No studies were identified on repeated use of COC ECPs. No studies were identified on key safety outcomes among study populations who might be at higher baseline risk, such as repeated use of COC ECPs and risk of thrombosis among women with underlying thrombotic conditions. No studies were identified that examined patient satisfaction with repeated use of ECPs. No studies reported occurrence of thrombosis, hospitalisation, haemorrhage or death; however, considering the rarity of such events, definitive conclusions cannot be drawn.

Future research should continue to expand this topic with more robust research methodologies, standardised dosages and direct comparison groups (eg, 30 mg UPA repeat dosing compared with LNG 1.5 mg repeat dosing compared with control or placebo) with further exploration of number of doses and specific time ranges or dosing windows.

ECPs are safe and can be used to prevent pregnancy after UPSI.^1^,^3^ Some women may find it necessary to use ECPs more than once in a given menstrual cycle. While data are limited and of very low certainty, the body of evidence does not suggest an increased risk of adverse events with repeated ECP use in a single menstrual cycle.

## Supplementary material

10.1136/bmjsrh-2025-202841 online supplemental file 1

## Data Availability

Data are available upon reasonable request.
