# Supplementary material for: Safety of repeated use of emergency contraceptive pills in the same menstrual cycle: a systematic review
Source: BMJ Sex Reprod Health. 2025 Nov 5;51(Suppl 1):e202841. doi: 10.1136/bmjsrh-2025-202841 (PMC12703341; doi:10.1136/bmjsrh-2025-202841)
Supplement: online supplemental file 1 [file bmjsrh-51-Suppl_1-s001.docx]

**Appendix 1. Search strategy**

| **Database** | **Strategy** |
| --- | --- |
| **Medline**  **(OVID)**  **1946-** | 1. exp Contraceptives, Postcoital/ 2. exp Contraception, Postcoital/  ((Contracept* ADJ5 (emergency OR postcoital OR post-coital OR postcoitus OR post-coitus OR pericoital)) OR (levonorgestrel ADJ5 (emergency OR postcoital OR post-coital OR postcoitus OR post-coitus OR pericoital)) OR ((contracept* OR postcoital OR post-coital OR postcoitus OR post-coitus OR pericoital) AND (ulipristal OR ella OR EllaOne OR UPA OR Plan B)) OR morning-after pill* OR yuzpe).ti,ab,kf.  1. 1 OR 2 OR 3 2. Dose-Response Relationship, Drug/ 3. ((Repeat* ADJ5 use*) OR (Repeat* ADJ5 dose*) OR (Repeat* ADJ5 dosing) OR (repeat ADJ5 dosage) OR (repeat* ADJ5 administ*) OR (repeat* ADJ5 treat*) OR (improper* ADJ5 use*) OR (proper* ADJ5 use*) OR (safe* ADJ5 use*) OR (unsafe* ADJ5 use*) OR (safe* ADJ5 dose*) or (unsafe* ADJ5 dose*) OR (dose* ADJ5 response*) OR (dose* ADJ5 administ*) OR (multi* ADJ5 dose*) OR (extra ADJ5 dose*) OR (double ADJ5 dose*) OR (two ADJ5 dose*) OR (first ADJ5 dose*) OR (second ADJ5 dose*) OR (single ADJ5 dose*) OR (one ADJ5 dose) OR (Three ADJ5 dose*) OR (third ADJ5 dose*) OR (four* ADJ5 dose*) OR (five ADJ5 dose*) OR (fifth ADJ5 dose*) OR (maxi* ADJ5 dose*) OR (number ADJ5 dose*) OR (allowed ADJ5 dose*) OR (subsequent* ADJ5 dose*) OR (initial ADJ5 dose*) OR (dose* AND (adverse event* OR adverse effect* OR adverse reaction* OR side effect* OR outcome*))).mp. 4. 5 OR 6 5. 4 AND 7 6. Exp animals/ NOT exp humans/ 7. 8 NOT 9 |
| **Embase**  **(OVID)**  **1947-** | 1. exp postcoitus contraceptive agent/  exp emergency contraception/((Contracept* ADJ5 (emergency OR postcoital OR post-coital OR postcoitus OR post-coitus OR pericoital)) OR (levonorgestrel ADJ5 (emergency OR postcoital OR post-coital OR postcoitus OR post-coitus OR pericoital)) OR ((contracept* OR postcoital OR post-coital OR postcoitus OR post-coitus OR pericoital) AND (ulipristal OR ella OR EllaOne OR UPA OR Plan B)) OR morning-after pill* OR yuzpe).ti,ab,kf.  1. 1 OR 2 OR 3 2. Drug dose/ OR drug dose comparison/ 3. ((Repeat* ADJ5 use*) OR (Repeat* ADJ5 dose*) OR (Repeat* ADJ5 dosing) OR (repeat* ADJ5 administ*) OR (repeat* ADJ5 treat*) OR (improper* ADJ5 use*) OR (proper* ADJ5 use*) OR (safe* ADJ5 use*) OR (unsafe* ADJ5 use*) OR (safe* ADJ5 dose*) or (unsafe* ADJ5 dose*) OR dose-response OR (dose* ADJ5 administ*) OR (multi* ADJ5 dose*) OR (extra ADJ5 dose*) OR (double ADJ5 dose*) OR (two ADJ5 dose*) OR (first ADJ5 dose*) OR (second ADJ5 dose*) OR (single ADJ5 dose*) OR (one ADJ5 dose) OR (Three ADJ5 dose*) OR (third ADJ5 dose*) OR (four* ADJ5 dose*) OR (five ADJ5 dose*) OR (fifth ADJ5 dose*) OR (maxi* ADJ5 dose*) OR (number ADJ5 dose*) OR (allowed ADJ5 dose*) OR (subsequent* ADJ5 dose*) OR (initial ADJ5 dose*) OR (dose* AND (adverse event* OR adverse effect* OR adverse reaction* OR side effect* OR outcome*))).ti,ab,kf. 4. 5 OR 6 5. 4 AND 7 6. Exp animal/ NOT exp human/ 7. 8 NOT 9 8. Limit 11 to "pubmed/medline" 9. 11 NOT 12 10. Limit 13 to conference abstract status 11. 13 NOT 14 |
| **Cochrane Library** | 1. [mh "Contraceptives, Postcoital"] 2. [mh "Contraception, Postcoital"] 3. ((Contracept*:ti,ab,kw NEAR/5 (emergency:ti,ab,kw OR postcoital:ti,ab,kw OR post-coital:ti,ab,kw OR postcoitus:ti,ab,kw OR post-coitus:ti,ab,kw OR pericoital:ti,ab,kw)) OR (levonorgestrel:ti,ab,kw NEAR/5 (emergency:ti,ab,kw OR postcoital:ti,ab,kw OR post-coital:ti,ab,kw OR postcoitus:ti,ab,kw OR post-coitus:ti,ab,kw OR pericoital:ti,ab,kw)) OR ((contracept*:ti,ab,kw OR postcoital:ti,ab,kw OR post-coital:ti,ab,kw OR postcoitus:ti,ab,kw OR post-coitus:ti,ab,kw OR pericoital:ti,ab,kw) AND (ulipristal:ti,ab,kw OR ella:ti,ab,kw OR EllaOne:ti,ab,kw OR UPA:ti,ab,kw OR "Plan B":ti,ab,kw)) OR ("morning-after" NEXT pill*):ti,ab,kw OR yuzpe:ti,ab,kw) 4. #1 OR #2 OR #3 5. [mh ^"Dose-Response Relationship, Drug"] 6. ((Repeat*:ti,ab,kw NEAR/5 use*:ti,ab,kw) OR (Repeat*:ti,ab,kw NEAR/5 dose*:ti,ab,kw) OR (Repeat*:ti,ab,kw NEAR/5 dosing:ti,ab,kw) OR (repeat:ti,ab,kw NEAR/5 dosage:ti,ab,kw) OR (repeat*:ti,ab,kw NEAR/5 administ*:ti,ab,kw) OR (repeat*:ti,ab,kw NEAR/5 treat*:ti,ab,kw) OR (improper*:ti,ab,kw NEAR/5 use*:ti,ab,kw) OR (proper*:ti,ab,kw NEAR/5 use*:ti,ab,kw) OR (safe*:ti,ab,kw NEAR/5 use*:ti,ab,kw) OR (unsafe*:ti,ab,kw NEAR/5 use*:ti,ab,kw) OR (safe*:ti,ab,kw NEAR/5 dose*:ti,ab,kw) OR (unsafe*:ti,ab,kw NEAR/5 dose*:ti,ab,kw) OR (dose*:ti,ab,kw NEAR/5 response*:ti,ab,kw) OR (dose*:ti,ab,kw NEAR/5 administ*:ti,ab,kw) OR (multi*:ti,ab,kw NEAR/5 dose*:ti,ab,kw) OR (extra:ti,ab,kw NEAR/5 dose*:ti,ab,kw) OR (double:ti,ab,kw NEAR/5 dose*:ti,ab,kw) OR (two:ti,ab,kw NEAR/5 dose*:ti,ab,kw) OR (first:ti,ab,kw NEAR/5 dose*:ti,ab,kw) OR (second:ti,ab,kw NEAR/5 dose*:ti,ab,kw) OR (single:ti,ab,kw NEAR/5 dose*:ti,ab,kw) OR (one:ti,ab,kw NEAR/5 dose:ti,ab,kw) OR (Three:ti,ab,kw NEAR/5 dose*:ti,ab,kw) OR (third:ti,ab,kw NEAR/5 dose*:ti,ab,kw) OR (four*:ti,ab,kw NEAR/5 dose*:ti,ab,kw) OR (five:ti,ab,kw NEAR/5 dose*:ti,ab,kw) OR (fifth:ti,ab,kw NEAR/5 dose*:ti,ab,kw) OR (maxi*:ti,ab,kw NEAR/5 dose*:ti,ab,kw) OR (number:ti,ab,kw NEAR/5 dose*:ti,ab,kw) OR (allowed:ti,ab,kw NEAR/5 dose*:ti,ab,kw) OR (subsequent*:ti,ab,kw NEAR/5 dose*:ti,ab,kw) OR (initial:ti,ab,kw NEAR/5 dose*:ti,ab,kw) OR (dose*:ti,ab,kw AND (("adverse" NEXT event*):ti,ab,kw OR ("adverse" NEXT effect*):ti,ab,kw OR ("adverse" NEXT reaction*):ti,ab,kw OR ("side" NEXT effect*):ti,ab,kw OR outcome*:ti,ab,kw))) 7. #5 OR #6 8. #4 AND #7 |
| **CINAHL**  **(EbscoHost)** | 1. (MH "Contraceptives, Postcoital+") 2. (((TI Contracept* OR AB Contracept* OR SU Contracept*) N5 ((TI emergency OR AB emergency OR SU emergency) OR (TI postcoital OR AB postcoital OR SU postcoital) OR (TI post-coital OR AB post-coital OR SU post-coital) OR (TI postcoitus OR AB postcoitus OR SU postcoitus) OR (TI post-coitus OR AB post-coitus OR SU post-coitus) OR (TI pericoital OR AB pericoital OR SU pericoital))) OR ((TI levonorgestrel OR AB levonorgestrel OR SU levonorgestrel) N5 ((TI emergency OR AB emergency OR SU emergency) OR (TI postcoital OR AB postcoital OR SU postcoital) OR (TI post-coital OR AB post-coital OR SU post-coital) OR (TI postcoitus OR AB postcoitus OR SU postcoitus) OR (TI post-coitus OR AB post-coitus OR SU post-coitus) OR (TI pericoital OR AB pericoital OR SU pericoital))) OR (((TI contracept* OR AB contracept* OR SU contracept*) OR (TI postcoital OR AB postcoital OR SU postcoital) OR (TI post-coital OR AB post-coital OR SU post-coital) OR (TI postcoitus OR AB postcoitus OR SU postcoitus) OR (TI post-coitus OR AB post-coitus OR SU post-coitus) OR (TI pericoital OR AB pericoital OR SU pericoital)) AND ((TI ulipristal OR AB ulipristal OR SU ulipristal) OR (TI ella OR AB ella OR SU ella) OR (TI EllaOne OR AB EllaOne OR SU EllaOne) OR (TI UPA OR AB UPA OR SU UPA) OR (TI "Plan B" OR AB "Plan B" OR SU "Plan B") OR (TI "morning-after pill*" OR AB "morning-after pill*" OR SU "morning-after pill*") OR (TI yuzpe OR AB yuzpe OR SU yuzpe)) 3. S1 OR S2 4. (MH "Dose-Response Relationship, Drug") 5. ((Repeat* N5 use*) OR (Repeat* N5 dose*) OR (Repeat* N5 dosing) OR (repeat N5 dosage) OR (repeat* N5 administ*) OR (repeat* N5 treat*) OR (improper* N5 use*) OR (proper* N5 use*) OR (safe* N5 use*) OR (unsafe* N5 use*) OR (safe* N5 dose*) OR (unsafe* N5 dose*) OR (dose* N5 response*) OR (dose* N5 administ*) OR (multi* N5 dose*) OR (extra N5 dose*) OR (double N5 dose*) OR (two N5 dose*) OR (first N5 dose*) OR (second N5 dose*) OR (single N5 dose*) OR (one N5 dose) OR (Three N5 dose*) OR (third N5 dose*) OR (four* N5 dose*) OR (five N5 dose*) OR (fifth N5 dose*) OR (maxi* N5 dose*) OR (number N5 dose*) OR (allowed N5 dose*) OR (subsequent* N5 dose*) OR (initial N5 dose*) OR (dose* AND ("adverse event*" OR "adverse effect*" OR "adverse reaction*" OR "side effect*" OR outcome*))) 6. S4 OR S5 7. S3 AND S6   **Limiters** - Exclude MEDLINE records |
| **Clinicaltrials.gov** | Completed Studies \| Emergency contraception OR emergency contraceptive OR postcoital contraception OR post-coital contraception OR postcoitus contraception OR post-coitus contraception OR pericoital contraception OR peri-coital contraception OR EllaOne OR "Plan B" OR "morning after pill" \| Studies with Female Participants |
